# Assessing the tolerability and efficacy of first-line chemotherapy in elderly patients with metastatic HER2−ve breast cancer

**DOI:** 10.3332/ecancer.2019.921

**Published:** 2019-04-03

**Authors:** Thomas Wilson, Claire Dyke, Hannah Reed, Zoe Hudson, Timothy Robinson, Paola Di Nardo

**Affiliations:** 1 Bristol Haematology and Oncology Centre, Horfield Road, Bristol BS2 8ED, UK; 2 Unit of Medical Oncology and Cancer Prevention, Centro di Riferimento Oncologico (CRO), IRCCS, Aviano, PN, Italy

**Keywords:** elderly patients, older adults, metastatic breast cancer, performance status, chemotherapy, tolerability, efficacy

## Abstract

**Background:**

In metastatic breast cancer (MBC), there is no consensus regarding the optimal regimen sequence and whether adults >65 years old (OA) are at increased risk from chemotherapy toxicity. Treatment decisions are often driven by the ability to tolerate treatment and maintain the quality of life. This study was designed to assess current practice in an oncology hospital in the UK.

**Methods:**

Retrospective data were collected about treatments used for 87 OA with MBC in a single centre between 2009 and 2016 to assess the tolerability and efficacy of first-line chemotherapy. Student’s T-tests and Kaplan-Meier statistical methods were applied.

**Results:**

70% of patients were commenced on standard dose (SD) of chemotherapy; 84% (21/25) of the anthracycline group (AG), 65% (20/31) of the capecitabine group (CG), 48% (10/21) of the taxane group (TG) and 100% (10/10) of other agents. 32% of patients had dose reductions; 16% in AG, 19% in TG and 58% in CG. Overall 30% of patients received six cycles of SD of chemotherapy; 36% in AG, 29% in CG and 14% in TG. 23% of patients suffered ≥grade 3 toxicity; 28% in AG, 29% in CG and 10% in TG. There were four treatment-related deaths; two in AG and one in both CG and TG. 61% of the CG received 6+ cycles with a mean on treatment time of 445 days (1–2,150). There was no statistical significance in progression- free survival (PFS) between groups. The median PFS for all patients was 244 days (87–381). Performance status, haemoglobin and estimated glomerular filtration rates prior to starting chemotherapy were all useful in predicting PFS.

**Conclusions:**

A relevant number of patients required dose reduction but dose-reduced chemotherapy was tolerated well. Anthracycline-based regimens were used in patients who had not received adjuvant chemotherapy. Capecitabine required the most dose reductions. Taxanes were generally started at reduced doses, resulting in fewer grade 3+ toxicities. As well as age, underlying physiological reserve, current performance status and co-morbidities should guide physicians who should consider lower starting doses in OA and recognise that dose reductions may be required to improve tolerability. The PFS of all regimens were similar in this study. This study highlights the need for further research to define the optimal first-line chemotherapy and starting dose in OA with MBC.

## Background

In metastatic breast cancer (MBC), there is no consensus regarding the optimal regimen sequence. Numerous clinical trials have been undertaken to try and establish which regimen should be used in the first-line metastatic setting. The table below summarises the outcomes of single and combination regimens used in the first-line setting to treat MBC (see Table 1).

The majority of first-line chemotherapy given in the metastatic setting is with regimens containing taxanes, capecitabine or, in patients who have not received them adjuvantly, anthracyclines. The progression-free survival (PFS) and overall survival of patients treated with anthracyclines, taxanes or capecitabine are broadly similar across studies [1-14]. Toxicities appear to increase in combination therapies. Taxanes, particularly Docetaxel, have higher rates of both febrile and non-febrile neutropenia [9–14]. Capecitabine has lower rates of haematological toxicity but higher rates of palmar-plantar erythema and in one study, high levels of fatigue [4]. Most studies do not comment on the number of patients requiring dose reductions or when therapy was stopped due to toxicity. However, in one capecitabine study, 16% of patients were stopped due to toxicity and a further 34% of patients had dose reductions [2]. It is clear that whilst taxanes, anthracyclines and capecitabine are efficacious in the first-line setting to treat patients with MBC, they all have potential toxicities that can require dose reductions or cessation of treatment. What remains unclear is whether any of the drugs are significantly better tolerated than the others.

In adults >65 years old (OA), there are numerous challenges to prescribing chemotherapy and dose reductions should be considered to increase tolerability. There are recognised physiological and psychosocial changes in OA, including declining renal function (reducing the rate of chemotherapy excretion), reduced bone marrow reserve (increasing the risk of haematological toxicity), pre-existing heart disease, reduced muscle mass and functional status, polypharmacy (affecting compliance and drug interaction), impaired cognition (affecting compliance and possibly causing treatment delays), malnutrition and psychological status and social support [15]. Renal insufficiency is a particular issue in OA, where serum creatinine, the standard measurements of renal function in most studies often underestimates renal impairment in OA due to their lower skeletal muscle mass. Creatinine clearance or estimated glomerular filtration rate (eGFR) is considered more reliable assessment methods [16].

OA have increased risk of chemotherapy toxicities. Treatment decisions are often driven by the ability to tolerate treatment and maintain the quality of life. There have been numerous studies of the effects of chemotherapy in elderly patients; the general consensus between these studies is that there does not appear to be any difference in the efficacy of chemotherapy in elderly patients compared to those less than 65 years old (see Table 2). However, OA experienced more toxicities, were often started on single rather than multiple agent regimens and lower percentages of patients completed all cycles of chemotherapy [4, 19–22].

There are various tools to assess vulnerability in OA prior to administering cancer therapies [23]. Karnofsky Performance Status and Eastern Cooperative Oncology Group (ECOG) performance status, whilst commonly used, have questionable predictive value regarding the risk of toxicity in OA [24]. The optimal approach is the Comprehensive Geriatric Assessment (CGA) which encompasses the main geriatric domains and can be used to help predict complications and side effects of treatment, in addition to estimating survival [25]. The CGA, however, can be considered time-consuming and therefore is not widely used in routine oncology clinical practice. The G8 validated screening tool, however, can be utilised in the first instance to identify those OA requiring a more detailed geriatric assessment and intervention to optimise this group pre-treatment and reduce toxicity prior to commencing treatment [23, 26].

This retrospective study compared different chemotherapy regimens to assess tolerability and efficacy in the treatment of >65-year-old patients being treated with first-line chemotherapy for metastatic human epidermal growth factor 2 (HER2)−ve breast cancer.

## Methods

Between 01/01/09 and 31/12/15 in a single tertiary oncology centre, data were collected on patients who were 65 years old or more at the time of starting first-line chemotherapy for MBC. Patients who were HER2+ve were not included, as these patients would receive anti-HER2 therapies in combination with chemotherapy, the addition of which would alter the toxicity and efficacy profile of these regimens. Paper and electronic notes were reviewed to confirm diagnosis and disease status at the time of chemotherapy. Chemotherapy prescriptions were reviewed to confirm the starting date, starting dose, any dose reductions and the date the regimen was last given.

Patients were grouped by the type of first-line chemotherapy they received.

Inclusion criteria:

- >65 years old at the time of starting chemotherapy
- Chemotherapy prescription for each cycle
- Oncology notes with receptor status, past medical history and drug history at the time of chemotherapy
- Blood results including haemoglobin and renal function
- Measurable disease of CT imaging
- Regular CT imaging to assess treatment response

Exclusion criteria:

- HER2+ve patients

To assess efficacy, the date of progression was taken as the first CT imaging showing progressive disease by Response Evaluation Criteria in Solid Tumours (RECIST) criteria. This was then used to calculate the PFS for each patient.

To assess tolerability, data were collected on the starting dose of chemotherapy and whether this was a standard or reduced dose, any dose reductions or delays that occurred during the regimen and any grade 3 or greater toxicities that occurred whilst on chemotherapy. The percentage of patients who received 100% of the intended dose and the percentage of patients who received 100% of the standard dose (SD) of each chemotherapy regimen were calculated.

Student’s *T*-test and Chi-squared tests were applied to the data sets to assess for significance. Kaplan-Meier curves were generated for PFS.

## Results

Eighty-seven patients > 65 years old (yo) were treated with first-line chemotherapy for metastatic HER2−ve breast cancer between 2009 and 2016. Seventy-five patients were oestrogen receptor positive (ER+) and 12 patients had triple-negative phenotype. The majority of patients (77/87) were treated with anthracyclines, capecitabine or taxanes. For ER+ patients, 83% had received previous anti-oestrogen therapy prior to commencing first-line chemotherapy for metastatic disease. The median age at the time of receiving chemotherapy was 72 years old; AG 74 yo, CG 72 yo and TG 71 yo. There was an even split of triple negative patients across the main regimens; 16% of the AG, 10% of the CG and 10% of the TG. Adjuvant chemotherapy had been given to 36% of patients. Of these, higher numbers received capecitabine or taxanes in the first-line metastatic setting (see Table 3).

70% of patients were commenced on SDs of chemotherapy, with higher percentages amongst the anthracycline patients (84%) over either the capecitabine group (65%) or the taxane group, where only 48% of patients were started on the SD. During chemotherapy, 28 patients received a dose reduction. This was highest amongst the capecitabine group where 58% of patients had at least one dose reduction. Eight patients received a second dose reduction, where six of these were in the capecitabine group. 30% of patients received six cycles of chemotherapy at the SD with the lowest percentages in the taxane group (14%). 62% of patients received at least three cycles of chemotherapy at the chosen starting dose; 64% in AG, 64.5% in CG and 62% in TG. Fifteen patients had delays of more than 2 weeks; seven of these were in the CG and three each in the AG and TG. 23% of patients had a toxicity graded 3 or more during chemotherapy. Higher percentages were seen in the AG (28%) and CG (29%) than the TG (10%). There were four on treatment deaths; two within the AG (one on day 7 and one on day 76) and one in each of the CG (on day 32) and TG (on day 90) (see Table 4 for summary).

**Graph 1.**
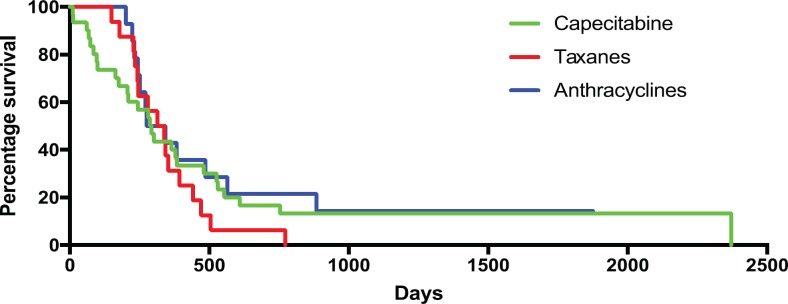
Kaplan-Meier curve of PFS by regimen

The second part of this study looked into treatment efficacy. 68% of patients had the best response of stable disease (36%) or partial response (PR) (32%) on CT scan by RECIST criteria. This was similar across the AG (68%), CG (71%) and TG (71%). This study defined clinical benefit as patients achieving either stable disease or a PR and receiving at least three cycles of chemotherapy at the intended dose. To achieve clinical benefit, an agent must be tolerable and efficacious. 52% of patients achieved clinical benefit with 22% achieving PR; 56% in the AG (24% PR), 51% in CG (19% PR) and 52% in TG (19% PR). The median PFS was 8.0 months (Interquartile range = 2.9–12.5 months). There were no significant differences in the PFS of the AG, CG and TG when compared to each other although there was a non-significant trend to suggest patients in the CG were performing better (see Table 5 and Graph 1 for summary).

This study also assessed the effect of performance status (PS) at the time of starting chemotherapy on PFS. Ten patients had a PS of 0 with a median PFS of 7.4 months, 39 patients had a PS of 1 with a median PFS of 9.1 months, 11 patients had a PS of 2 with a median PFS of 2.2 months and one patient had a PS of 3 with a PFS of 0.2 months. The effect of poor pre-morbidity of PFS was assessed by comparing patients with adequate and inadequate haemoglobin and creatinine clearance. Seventy patients had an eGFR of ≥34 mls/min/1.73 m^2^, the median PFS of these patients was significantly better at 8.8 months compared to 3.1 months in the 17 patients with an eGFR <34 mls/min/1.73 m^2^ (*p* = 0.004). Seventy-two patients had haemoglobin of ≥100 g/L, the median PFS of these patients was significantly better at 8.5 months compared to 3.2 months in the 15 patients with haemoglobin of <100 g/L (*p* = 0.004) (see Table 6 for summary).

## Discussion

There is no clear consensus as to the tolerability of chemotherapy in OA with MBC. 23% of patients in this study experienced grade 3–5 toxicity from treatment, 10% of these were haematological and 90% were non-haematological. The most common grade 3+ toxicities were plantar palmar erythema (25%) and fatigue (20%). There were two episodes of neutropenic sepsis both in the AG. A previous breast cancer study assessing similar treatments showed 17% of over 65-year-old patients experiencing grade 3–5 toxicity [18]. Conversely, in another study of 500 over 65-year-old patients in multiple different cancers, 53% of patients experienced grade 3–5 toxicity [18]. In this study, there were four on treatment deaths, two of these occurred in the AG, this might reflect the higher percentage of the AG started on SDs of chemotherapy (84%). Similarly, more patients in the CG and AG suffered grade 3–5 toxicities (29% and 28%, respectively) than in the TG (10%). The apparent better tolerance of taxanes is probably because 52% of patients were started on a lower than standard treatment dose. The CG had a much higher number of dose reductions (58% CG versus 16% AG and 19% TG) but it is used on a continuous dosing strategy as opposed to limited to six cycles as for anthracyclines and taxanes and therefore there are more opportunities for toxicities to either occur or progress. 44% of the CG dose reductions occurred after patients had already received six cycles. Almost two-thirds of patients in all three treatment groups received a minimum of three cycles at the starting dose chosen. This suggests that in the majority of cases the correct starting dose was chosen.

The majority of the TG were started on lower than SDs; however, the PFS of these patients was equal to historical controls of younger patients treated at full dose. This group had the least grade 3–5 toxicity and the number of 2 week delays. Dose reductions in the TG group were comparable to the AG and were better than the CG. In the AG, most patients were chemotherapy-naive, perhaps making it more difficult to assess the likelihood tolerating SDs; consequently, the majority were started on SDs. The least number of dose reductions were undertaken in this group and these patients also had the least number of delays greater than 2 weeks.

The efficacy of taxanes, anthracyclines and capecitabine in this study was very similar. The number of patients achieving stable disease and PRs on CT imaging by RECIST criteria was comparable across the groups. The median PFS for all groups was generally better than those seen in previous studies [1–14]. In this study, the median PFS for capecitabine (9.2 months) was better than the PFS seen in previous studies (4.1 to 7.7 months) [1-5]. The median PFS for taxanes (7.8 months) was in keeping with previous studies (3.6–12.9 months) [5, 9–14]. Similarly, the median PFS for anthracyclines (8 months) was in keeping with previous studies (3.1–11 months) [5–10]. There is a trend in this study to suggest that the PFS is better with capecitabine, possibly secondary to its use as a continuous treatment rather than for a maximum of six cycles as for taxanes and anthracyclines.

This study supports previous literature in suggesting there are no significant differences between the PFS using taxanes, anthracyclines or capecitabine in the first-line setting for patients with metastatic HER2−ve breast cancer. It also supports previous studies suggesting there is no difference in the efficacy of chemotherapy when used in elderly patients [4, 16–21]. The PFS of all regimens in this study is on par with those seen in younger patients.

The PFS of patients was affected by their pre-morbid condition. There was a trend to suggest patients with a better performance status by ECOG assessment before starting chemotherapy had longer PFS. Patients with adequate renal function and haemoglobin had significantly longer PFS. This reflects the greater reserves these patients have to cope with chemotherapy due to the lower physiological burden of disease and any underlying co-morbidities.

## Conclusion

Whilst results from this retrospective study must be interpreted with caution as it only reflects a single centre’s experience and includes fewer patients than some of the other studies discussed, the efficacy and tolerability of first-line chemotherapy in OA with metastatic HER2−ve breast cancer appears to be comparable to younger patients. There was no statistical difference in the PFS of anthracyclines, capecitabine or taxanes in this study. Dose reductions and lower starting doses should be considered to improve tolerability as reduced doses do not appear to affect efficacy. Chronological age alone should not be used as a determinant of treatment decisions but it should instead prompt a more comprehensive review of a patient’s functional status, co-morbidities, polypharmacy, support networks, expectations, preferences and life expectancy. The use of geriatric assessments such as CGA can be useful in some patients to aid management decisions.

## Conflicts of interest

The authors do not have any conflicts of interest to declare.

## Funding statement

Timothy Robinson received funding from Amgen and Daiichi-Sankyo to attend educational workshops. All other authors have no financial declarations.

## Figures and Tables

**Table 1.** Summary of previous studies into first-line chemotherapy for MBC.

	Regimen	No. of patients	PFS (months)	OS (months)	Toxicity
Fumoleau *et al* [1]	Capecitabine	126	4.9	15.2	
O’Shaughnessy *et al* [2]	Capecitabine	95	4.1	19.6	16% patients stopped due to toxicity 34% dose reduced
Stockler* et al* [3]	Capecitabine	323	6	22	
Smorenburg *et al* [4]	Capecitabine	78	7.7	16.8	G3 fatigue 13%, G3 palmar plantar erythema 16%, G3 Diarrhoea 5%
Robert *et al* [5]	Capecitabine Taxane Anthracycline	615 307 315	5.7 8 8		
Focan *et al* [6]	Epirubicin	141	8		
Harris *et al* [7]	Doxorubicin	224	3.1	20	
Chan *et al* [8]	Doxorubicin + Cyclophosphamide Epirubicin + Cyclophosphamide	160	7.7 5.6	18.3 16	
Biganzoli *et al* [9]	Doxorubicin + Paclitaxel Doxorubicin + Cyclophosphamide	275	6 6	20.5 20.6	32% febrile neutropenia in Dox./Pac. arm
Pacilio *et al* [10]	Epirubicin Epirubicin + Docetaxel	51	9 11	18 21	Increased toxicity in Epi./Doc. arm
Gradishar *et al* [11]	Nab-Paclitaxel Docetaxel	302	12.9 7.5		G4 neutropenia 75% Doc., 39% nab-Pac. G3 fatigue 19% Doc. arm
Takashima *et al* [12]	Taxanes	618		37.2	G3 neutropenia 3%, G3 fatigue 4%
Albain *et al* [13]	Gemcitabine + Paclitaxel Paclitaxel	529	6.1 4	18.5 15.8	6.7% Gem./Pac. and 5% Pac. stopped due to toxicity
Jones *et al* [14]	Docetaxel Paclitaxel	449	5.7 3.6	15.4 12.7	Increased haematological and non-haematological toxicity in Doc. arm

**Table 2.** Summary of studies investigating the efficacy and toxicity seen in OA treated with chemotherapy.

	Cancer type and patients included (age in years)	No. of patients	Survival outcomes		Toxicity outcomes
Smorenburg* et al* [4]	Breast >65	78	65–75 years old no differences		1. 65–75 years old no differences 2. >80 years old: Only one in ten patients completed chemotherapy
Begg *et al* [17]	Lung Breast Colorectal <65 versus >65	3,000 (Meta-analysis)	Equivalent response rates between <65 and >65 patients		1. No increase in frequency or severity of toxicity in >65 years old versus <65 years old 2. Compliance similar between both groups
Christman *et al* [18]	Breast <50 50–65 >65	40 60 70	PFS	OS	1. Toxicity frequency and severity same across groups 2. Same number of dose delays seen across groups
			9.1 months	17.9 months	
			6.2 months	12.8 months	
			7.2 months	14.2 months	
Crivellari* et al* [19]	Breast <65 versus >65	299	Same PFS between groups		1. G3 toxicity 17% in >65 years old versus 7% in <65 years old 2. More of >65 years old group received less than expected doses
Hurria* et al* [20]	Lung, Gastrointestinal, Breast, Gynae Genitourinary >65	500	Not assessed		1. 53% of patients experienced G3–5 toxicity with 2% grade 5 toxicity
Extermann* et al* [21]	Any Cancer >70	518	Not assessed		1. 64% of patients experienced severe toxicity
Lund* et al* [22]	Colorectal <70 versus >70	529	1. No difference in age-adjusted disease-free survival (DFS) or 10 years mortality 2. Poor performance status across all ages led to worse DFS and 10 years mortality rates		1. No age-adjusted difference in G3–5 toxicity 2. Elderly patients more frequently received single versus doublet chemotherapy

**Table 3.** Patient demographics and usage of previous chemotherapy and endocrine therapy in management. Bracketed figures are percentage values.

Regimen	Number	Age <75	Age 75+	ER+	Triple−ve	Adjuvant chemo	Previous anti-oestrogen
Total patients	87	56 (64)	31 (36)	75 (82)	12 (18)	31 (36)	72 (83)
Anthracyclines	25	11 (44)	14 (56)	21 (84)	4 (16)	2 (8)	21 (84)
Capecitabine	31	22 (71)	9 (22)	28 (90)	3 (10)	13 (42)	27 (87)
Taxanes	21	15 (71)	6 (29)	19 (90)	2 (10)	12 (57)	18 (86)
Mix	7	5 (71)	2 (29)	6 (86)	1 (14)	2 (29)	5 (71)
Vinca Alkaloids	2	2 (100)	0 (0)	1 (50)	1 (50)	1 (50)	1 (50)
Platinum	1	1 (100)	0 (0)	0 (0)	1 (100)	1 (100)	0 (0)

**Table 4.** Summary of chemotherapy tolerability. Bracketed figures are percentage values.

Regimen	Number	SD chemotherapy commenced	Dose reductions	Six cycles SD received	Three cycles of intended dose chemotherapy received	2 week+ delays	≥grade 3 toxicity	Deaths
Total Patients	87	61 (70)	28 (32)	26 (30)	54 (62)	15 (17)	20 (23)	4 (5)
Anthracyclines	25	21 (84)	4 (16)	9 (36)	16 (64)	3 (12)	7 (28)	2 (8)
Capecitabine	31	20 (65)	18 (58)	9 (29)	20 (64.5)	7 (23)	9 (29)	1 (3)
Taxanes	21	10 (48)	4 (19)	3 (14)	13 (62)	3 (14)	2 (10)	1 (4)
Others	10	10 (100)	2 (29)	5 (50)	5 (50)	2 (20)	2 (29)	0 (0)

**Table 5.** Efficacy of chemotherapy regimens. Bracketed figures are percentage values.

Regimen	Best response on CT		Clinical benefit rates		Progression-free survival (months)		
	SD	PR	SD + 3C chemo	PR + 3C chemo	Mean	Median	Interquartile range
Total patients	31 (36)	28 (32)	26 (30)	19 (22)	9.6	8.0	2.9–12.5
Anthracyclines	9 (36)	8 (32)	8 (32)	6 (24)	8.4	8.0	2.6–11.6
Capecitabine	12 (39)	10 (32)	10 (32)	6 (19)	11.7	9.2	3.2–15.8
Taxanes	8 (38)	7 (33)	7 (33)	4 (19)	8.8	7.8	3.0–11.3
Others	2 (20)	3 (30)	1 (10)	3 (30)	5.4	4.9	2.0–9.1

**Table 6.** Summary of effect of performance status, haemoglobin and renal function on PFS.

		Patient number	Median progression-free survival (months)	Interquartile range (months)	Significance (*p* values)
Performance status	0	10	7.4	3.5–17.1	No significance seen
	1	39	9.1	3.7–14.3	
	2	11	2.2	1.2–2.9	
	3	1	0.2		
Estimated GFR ≥34 mls/min/1.73 m^2^	Yes	70	8.8	3.7–15.9	0.004
	No	17	3.1	2.2–8.3	
Haemoglobin ≥100 g/L	Yes	72	8.5	3.0–15.7	0.004
	No	15	3.2	2.6–9.1	
